# NMR-based metabolomics identification of potential serum biomarkers of disease progression in patients with multiple sclerosis

**DOI:** 10.1038/s41598-024-64490-x

**Published:** 2024-06-26

**Authors:** Mohammad Alwahsh, Refat M. Nimer, Lina A. Dahabiyeh, Lama Hamadneh, Aya Hasan, Rahaf Alejel, Roland Hergenröder

**Affiliations:** 1 Faculty of Pharmacy, Al-Zaytoonah University of Jordan, Amman, 17138 Jordan; 2 Department of Medical Laboratory Sciences, Jordan University of Science and Technology, Irbid, 22110 Jordan; 3 Department of Pharmaceutical Sciences, School of Pharmacy, The University of Jordan, Amman, 11942 Jordan; 4 Department of Badic Medical Sciences, Faculty of Medicine, Al-Balqa Applied University, Al-Salt, Jordan; 5 Leibniz-Institut Für Analytische Wissenschaften-ISAS-E.V., 44139 Dortmund, Germany

**Keywords:** Multiple sclerosis, Metabolomic, Nuclear magnetic resonance spectroscopy, Relapsing remitting, Biomarker, Biomarkers, Diseases

## Abstract

Multiple sclerosis (MS) is a chronic and progressive neurological disorder, characterized by neuroinflammation and demyelination within the central nervous system (CNS). The etiology and the pathogenesis of MS are still unknown. Till now, no satisfactory treatments, diagnostic and prognostic biomarkers are available for MS. Therefore, we aimed to investigate metabolic alterations in patients with MS compared to controls and across MS subtypes. Metabolic profiles of serum samples from patients with MS (n = 90) and healthy control (n = 30) were determined by Nuclear Magnetic Resonance (^1^H-NMR) Spectroscopy using cryogenic probe. This approach was also utilized to identify significant differences between the metabolite profiles of the MS groups (primary progressive, secondary progressive, and relapsing–remitting) and the healthy controls. Concentrations of nine serum metabolites (adenosine triphosphate (ATP), tryptophan, formate, succinate, glutathione, inosine, histidine, pantothenate, and nicotinamide adenine dinucleotide (NAD)) were significantly higher in patients with MS compared to control. SPMS serum exhibited increased pantothenate and tryptophan than in PPMS. In addition, lysine, myo-inositol, and glutamate exhibited the highest discriminatory power (0.93, 95% CI 0.869–0.981; 0.92, 95% CI 0.859–0.969; 0.91, 95% CI 0.843–0.968 respectively) between healthy control and MS. Using NMR- based metabolomics, we identified a set of metabolites capable of classifying MS patients and controls. These findings confirmed untargeted metabolomics as a useful approach for the discovery of possible novel biomarkers that could aid in the diagnosis of the disease.

## Introduction

Multiple sclerosis (MS) is the most common chronic inflammatory, demyelinating, and neurodegenerative disease of the central nervous system (CNS) in young adults, mostly aged between 20 and 40, with female to male ratio of 3:1^1,2^. This condition is characterized by a combination of genetic and environmental factors affecting approximately 2.8 million individuals worldwide^3^. MS encompasses four subtypes: clinically isolated syndrome (CIS), relapsing remitting MS (RRMS), primary progressive MS (PPMS), and secondary progressive MS (SPMS).

CIS refers to the initial clinical manifestation of a disease that exhibits inflammatory demyelination features, potentially indicative of MS. However, at this stage, it has not met the criteria for temporal dissemination^4^. RRMS represents the predominant subtype, accounting for approximately 85% of all clinically diagnosed cases of MS^5^. It is characterized by episodes of disease activity and symptom exacerbation followed by periods of symptom relief^6^. Over a span of 20 years, more than 70% of individuals with RRMS progress to SPMS^7^. While SPMS and PPMS share the onset, SPMS symptoms continue to worsen over time. On the other hand, PPMS symptoms steadily deteriorate without any improvement^8^. PPMS is considered a form of the disease where symptoms progressively worsen over time.

Despite the extensive research conducted over the years, there remains a notable lack of definitive diagnostic tests for confirming the presence of MS. Moreover, the phenotypes of MS cannot be differentiated from one another^9^. The current diagnostic procedure predominantly relies on a comprehensive patient medical history assessment, thorough physical examination, and magnetic resonance imaging^10^. In addition, individuals who have been diagnosed with MS frequently experience a wide range of symptoms. The symptoms reported encompass challenges in coordination, cognitive impairments, and visual loss^11,12^. The multifaceted underlying factors that contribute to MS may provide an explanation for the wide range of symptoms that are observed in affected individuals.

Metabolomics is a high-throughput approach to investigate a collection of small molecules with a mass typically between 50 and 1500 Daltons, such as amino acids, lipids, and carbohydrates^13^. Global metabolomics provides valuable insights into metabolic processes, improving our understanding of metabolic alterations that might be associated with the development and the progression of various diseases^14,15^. It plays a crucial role in the identification of biomarkers^16^. Furthermore, metabolomics differs from other omics measurements because it directly represents the phenotype by identifying metabolites and their concentrations^17^.

Recent advances in technology in nuclear magnetic resonance (NMR) spectroscopy and mass spectrometry, which are used to profile metabolites^18^, have made analytical tests more sensitive and clearer in terms of spectral resolution. Even though Cryogenic ^1^H NMR might be less sensitive than mass spectrometry, it makes it easier to measure metabolite signals since minimal sample preparation is needed, the method is not invading and the use of standards for each compound is not required^19^. The use of NMR spectroscopy in metabolomics offers many advantages when it comes to reproducibility, sample recovery and analysis time, yet it still faces one major limitation concerning sensitivity compared to the high sensitivity of MS spectroscopy. Recent developments including the use of cryogenic and micro probes can help extend NMR sensitivity to overcome this problem^20,21^. However, the lack of sensitivity compared to mass-spectrometry is partly relativized by analytical performance in terms of ease of quantification, reproducibility, and analytical quality of data. Given its universal detection capability, q HNMR offers an unbiased overview of the sample composition^22^. Quantification in mass-spectrometry relies on calibration curves for each molecular species, in NMR spectroscopy only a single standard for all molecular species in the sample is used to calibrate for instrumental proton sensitivity. Especially in eucaryotic cells where only less than 100 primary metabolites are typically detected the annotation is not problematic, to identify ambiguous cases different parts of the spectra can be used or a plethora of two-dimensional NMR techniques is available. Therefore, sample preparation can be reduced to a simple extraction^23^ with easy to control carryover or contamination problems. Quality control is assured by regular sensitivity and frequency calibration with appropriate NMR calibration and reference standards. Care must be taken to assure that spin relaxation of all compounds has taken place between consecutive pulses. However, due to small sample size extra measures of quality control have not been taken^24,25^.

The etiology and the underlying pathophysiological disturbances linked to MS have not yet been fully understood. Besides, definitive diagnostic and prognostic biomarkers that can facilitate the detection and the differentiation of various subtypes of MS are still lacking. Therefore, in this study, we used Cryogenic ^1^H NMR spectroscopy metabolomics approach to identify novel biomarkers for MS disease.

## Material and methods

### Ethical considerations

This study was approved by the Institutional Review Board (IRB) (IRB no. 4718, 21/3/2022), et al.-Bashir Hospital, Amman, Jordan. Patients and healthy participants were asked to sign consent forms before taking part in this study.

### Subjects and selection criteria

This study included a total of 90 individuals who visited both private and public neurology clinics and were diagnosed with MS. The inclusion criteria for the study encompassed individuals aged 18 to 70 years with confirmed diagnosis of MS by a neurologist in accordance with the revised McDonald criteria^26^. MS severity was determined by the use of the Expanded Disability Status Scale (EDSS)^27^. The study was conducted in accordance with the Declaration of Helsinki, and approved by the institutional review board of the Jordan University of Science and Technology. Moreover, participants had provided informed consent before participating in the study. The study excluded individuals who had been diagnosed with chronic conditions other than MS. The participants were divided into three categories based on their type of MS; RRMS, PPMS, and SPMS.

Furthermore, we recruited 30 healthy control adults from the community with no signs of MS or other chronic diseases. The control group matched the MS group regarding age, gender, body mass index (BMI), and ethnic background.

### Sample collection, processing, and storage

A total of 10 mL of blood was collected from each participant in the study. Clot activator-free tubes (AFCO, Jordan) were used for the sample collection. Approximately 5.0 mL serum was then obtained by centrifuging the blood samples at 3000 g for 10 min. Serum samples were collected and stored at − 80 °C for further analysis.

### Metabolite extraction

Metabolite extraction was performed as previously described^28^. Briefly, to 35 µL serum, 345 µL cold LC–MS-grade methanol (Fisher Scientific, USA) was added followed by the addition of 172.5 µL LC–MS-grade chloroform and 88 µL LC–MS water (Fisher Scientific, USA). Equal volumes of chloroform and water were added followed by centrifugation at 10,000 × g for 5 min. From the upper separated phase, 400 µL was transferred to a new Eppendorf tube and sample was dried using vacuum centrifugal evaporator (Eppendorf, Hamburg, Germany) and stored at − 80 °C until further analysis.

### Sample preparation and measurement conditions for NMR

All NMR experiments for ^1^H detection was performed at 600.13 MHz on a BRUKER AVANCE NEO 600 spectrometer equipped with a cryogenic NMR probe (CP QCI 600S3 H-P/C/N-D 05-Z, 5 mm tubes) to improve sensitivity. Water suppression was achieved using a double watergate sequence with excitation sculpting^29^. The sample spectra were quantified using the "Electronic REference To access In vivo Concentrations" (ERETIC) technique^30^. The pulsprogram used in this study is ZGESGP (Buker name). Using TopSpin 4.0.7, the signal was achieved via an acquisition time of 1.38 s with 335 number of scans and relaxation delay of 4 s at 280 K for all samples. The extract was mixed with 600μL D2O, pipetted into a 5 mm NMR tubes (Boro-600-5-8, Deutero, Germany) and immediately used for NMR measurement.

### Data analysis

Metabolites identification and quantification was performed within Chenomx NMR suite v9.0 PRO (Chenomx Inc., Edmonton, AB, Canada). For this technique a 9 mmol/L aqueous sucrose solution was used as an external reference to calibrate the ERETIC2 reference for absolute concentration determination. Based on this sucrose reference, RF-signal was deliberately transmitted into the 1H spectrum of each sample. This artificial resonance was used to calibrate the deconvolution software Chenomx NMR suite. The advantage of this procedure is that the externally calibrated electronic resonance is free from matrix effects e.g. reported for TSP. All concentrations were reported in μM. MetaboAnalyst v5.0 (Xia Lab @ McGill university, Montreal, QC, Canada)^31^ was used to perform Orthogonal Partial Least Squares-Discriminant Analysis (OPLS-DA), Partial Least Squares-Discriminant Analysis (PLS-DA), heat map, pathway analysis and Variables Importance of Projection (VIP) to identify the metabolites that contribute to group separation. MetaboAnalyst software was used to evaluate model robustness using Receiver Operating Characteristic—Area Under Curve (ROC-AUC) analysis. Statistical significance was set at p < 0.05 (based on t-test and one-way ANOVA). The Rstudio software's "geom_boxplot" and "facet_wrap" modules were used to create the boxplots of metabolite concentrations. In addition, to create the volcano plot by using Rstudio software.

## Results

### Demographic and clinical data of participants

The data in Table 1 shows no significant differences observed between the control group and patients with MS in terms of age, BMI, and gender. Similarly, Table 2 illustrates that patients with subtypes of MS (RR, PP, and SP) were matched with regards to age, BMI, and gender.

**Table 1:** Demographic data of recruited control and patients with MS.

Demographic and clinical characteristics	MS (*n* = 90)		Control (*n* = 30)		p-value^b^
	Mean	SD	Mean	SD	
Age (years)	36.1	8.9	35.6	2.5	0.77
Gender (F/M)^a^	49/41	NA	15/15	NA	0.67
BMI (kg/m^2^)	26.9	6.5	28.3	2.2	0.24

*MS* multiple sclerosis; *F/M* female and male, *BMI* body mass index

^a^Presented as the number of subjects in each group. Values are presented as mean ± SD

^b^p-value using Student’s *t*-test for age and BMI and chi-square test for gender

**Table 2:** Demographic data of recruited patients with MS at RR, PP, and SP subtypes.

Demographic and clinical characteristics	RR-MS (*n* = 30)	PPMS (*n* = 30)	SPMS (*n* = 30)	p-value^b^
Age (years)	37.0 ± 8.6	33.4 ± 7.0	37.9 ± 10.5	0.12
Gender (F/M)^a^	19/11	15/15	15/15	0.49
BMI (kg/m^2^)	30.0 ± 5.8	24.9 ± 3.9	28.2 ± 8.0	0.20

*MS* multiple sclerosis, *RR* relapsing remitting, *PP* primary progressive, *SP* secondary progressive, *F*/*M* female and male, *BMI* body mass index

^a^Presented as the number of subjects in each group. Values are presented as mean ± SD

^b^p-value using one-way ANOVA test for age and BMI and chi-square test for gender

### ^1^H-NMR data of serum samples in patients with MS vs. control

A total of 40 metabolites were identified after ^1^H NMR spectroscopy analysis of 120 serum samples from MS patients and control subjects, the p-values illustrated in Table S1. Figure S2 depicts the levels of these metabolites. A representative ^1^H-NMR spectrum for healthy sample is shown in Figure S1.

### Metabolic profiling in patients with MS compared to healthy control

**Figure 1:**
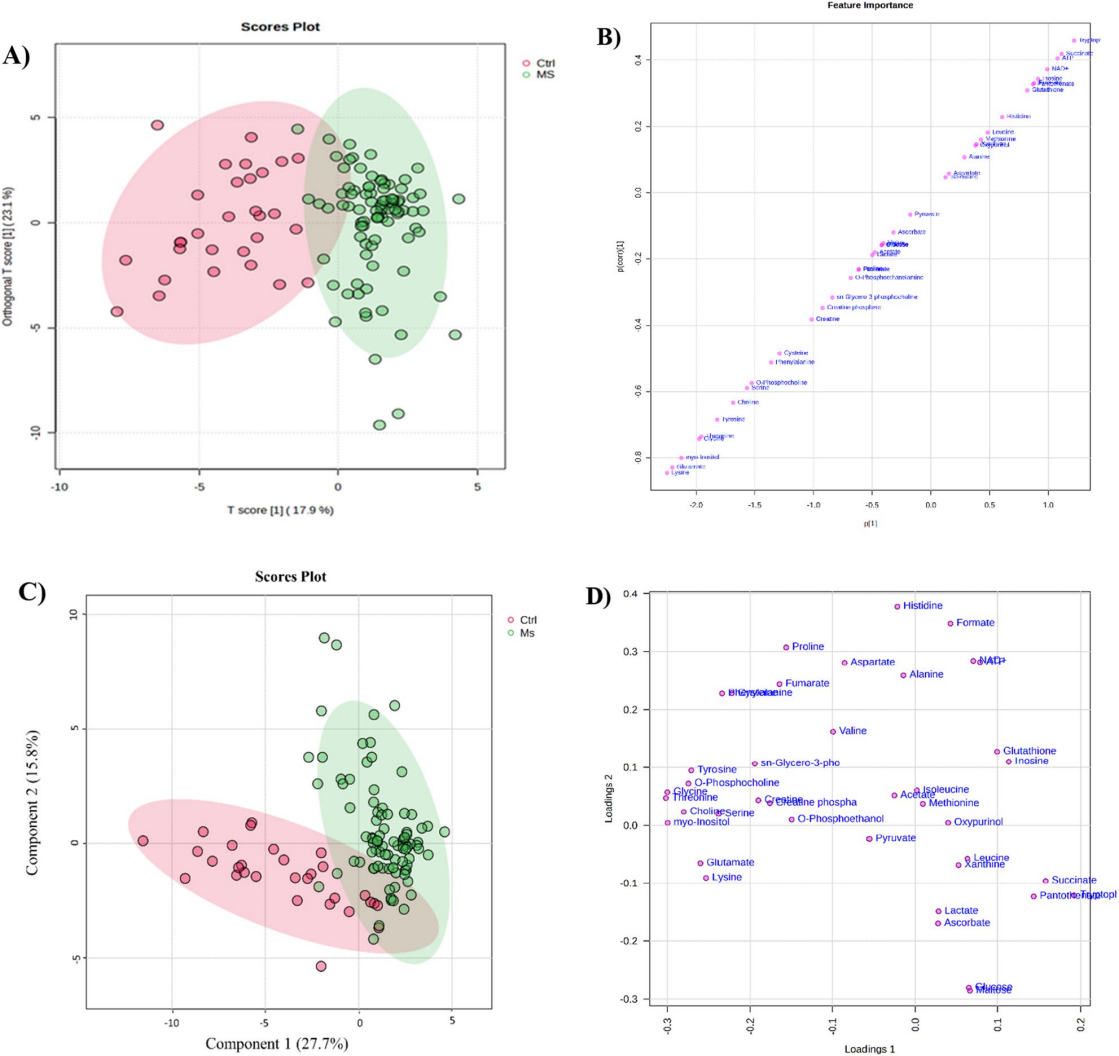
As multivariate analysis, (**A**) OPLS-DA score plot, (**B**) S-plot of the OPLS-DA model, (**C**) PLS-DA score plot and (**D**) Loading plot of PLS-DA model based on metabolic data between serum samples of patients with MS (n = 90) and healthy control (n = 30).

Univariate and multivariate analyses were employed to investigate the metabolites in serum that exhibited significant alterations between the MS group and the control group. A comparative analysis was conducted using orthogonal partial least squares discriminatory analysis (OPLS-DA) and Partial Least Squares Discriminant Analysis (PLS-DA). The results revealed a distinct clustering and a separation of the two study groups, indicating a metabolic difference between MS patients and controls (Fig. 1). The analysis of OPLS-DA model (R2X = 0.179, R2Y = 0.553 and Q2 = 0.526) yielded satisfactory R2Y and Q2 > 0.5. Additionally, to further examine clustering between control and MS group a PLS-DA score plot was generated. PLS-DA analysis yielded R2 = 0.837 and Q2 = 0.635 and showed a clear separation between the two groups. Lysine, glutamate, myo-Inositol, glycine, threonine, tyrosine, choline, serine, O-phosphocholine, phenylalanine, cysteine, and tryptophan represent the highest VIP scores more than 1 based on.

OPLS-DA model as presented in Figure S3.

**Figure 2:**
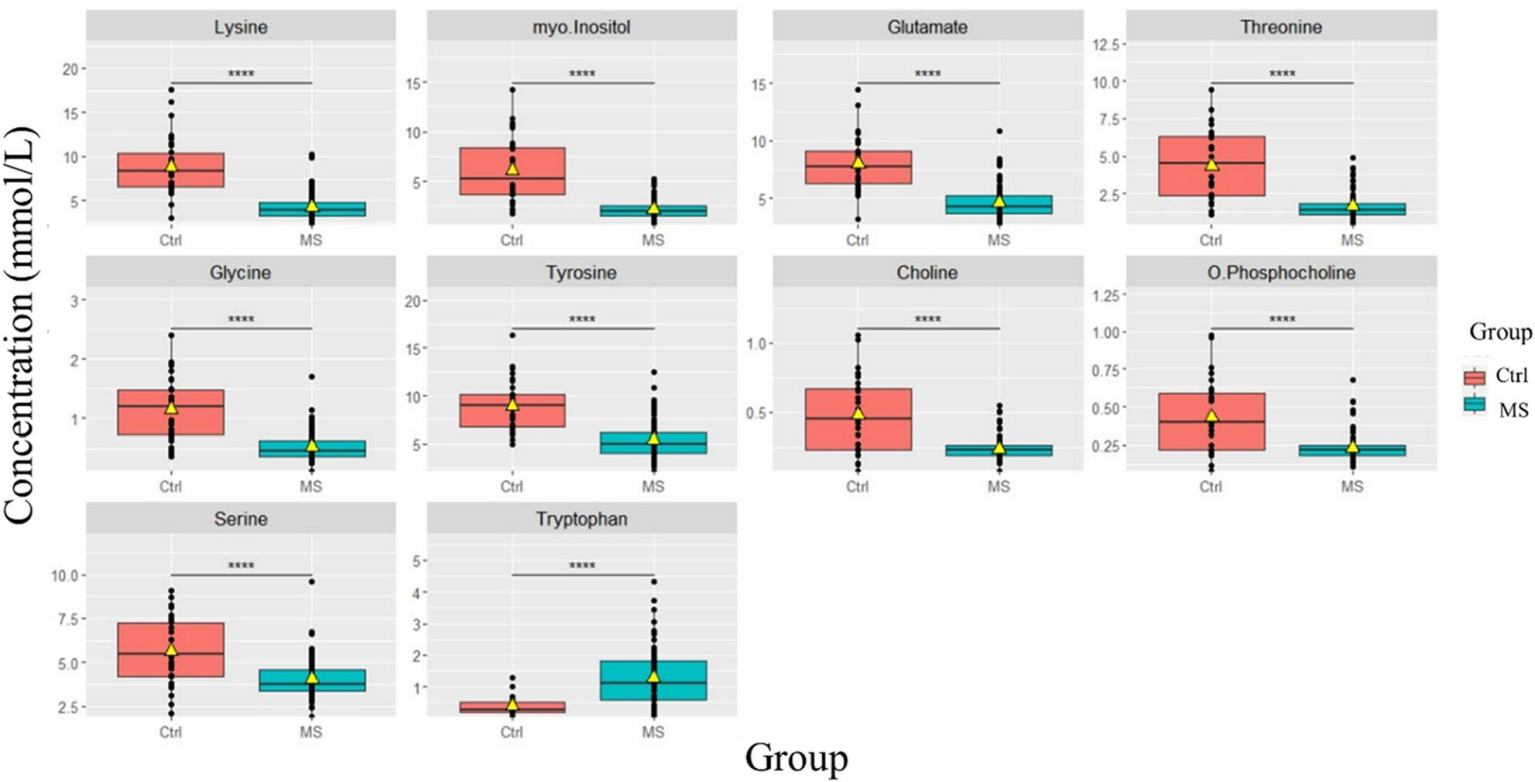
Box plots for the most significantly altered metabolites between healthy control (n = 30) and MS groups (n = 90) analyzed by t-test, p-value < 0.05. (****p < 0.0001).

All significant differences in metabolite concentrations presented in bold by the p-value in Table S1 and the top 10 significant metabolites in the boxplots (Fig. 2) were observed for comparisons of MS versus control groups. Tryptophan, succinate, adenosine triphosphate (ATP), formate, inosine, histidine, glutathione, and pantothenate were significantly higher in MS compared to control group. In contrast, lysine, myo-inositol, glutamate, threonine, glycine, tyrosine, choline, O-phosphocholine, serine, cysteine, and sn-glycero-3-phosphocholine were found to be significantly decreased in the serum of MS group compared with control as summarized in Table 3 and seen in Fig. S4.

**Table 3:** Change in the levels of significantly altered metabolites (n = 25) in MS group (n = 90) compared to control group (n = 30).

Metabolites	p-value^a^	Fold change (MS vs Ctrl)^b^
Lysine	3.11E-19 ****	↓ (0.48)
myo-Inositol	6.13E-18 ****	↓ (0.36)
Glutamate	1.56E-16 ****	↓ (0.57)
Threonine	7.64E-16 ****	↓ (0.38)
Glycine	9.51E-15 ****	↓ (0.45)
Tyrosine	3.33E-12 ****	↓ (0.61)
Choline	5.18E-12 ****	↓ (0.50)
O-phosphocholine	1.79E-09 ****	↓ (0.54)
Serine	7.47E-08 ****	↓ (0.71)
Tryptophan	4.39E-07 ****	↑ (3.34)
Phenylalanine	9.54E-07 ****	↓ (0.64)
Cysteine	1.10E-06 ****	↓ (0.65)
Succinate	2.50E-06 ****	↑ (1.58)
Creatine	1.64E-05 ****	↓ (0.46)
ATP	1.11E-04 ***	↑ (1.98)
Creatine phosphate	1.39E-04 ***	↓ (0.49)
NAD +	1.77E-04 ***	↑ (1.90)
O-phosphoethanolamine	4.79E-04 ***	↓ (0.70)
Formate	5.65E-04 ***	↑ (2.47)
Inosine	1.24E-03 **	↑ (1.61)
sn-glycero-3-phosphocholine	1.67E-03 **	↓ (0.77)
Histidine	2.90E-03 **	↑ (1.90)
Glutathione	3.29E-03 **	↑ (1.47)
Pantothenate	5.54E-03 **	↑ (1.37)
Fumarate	1.76E-02 *	↓ (0.57)

^a^p-values obtained by t-test, *p < 0.05, **p < 0.01, ***p < 0.001, and ****p < 0.0001.

^b^Metabolites with ↑ indicates increased levels in serum of MS group while ↓ indicates decreased levels in serum of MS group compared to control group.

**Figure 3:**
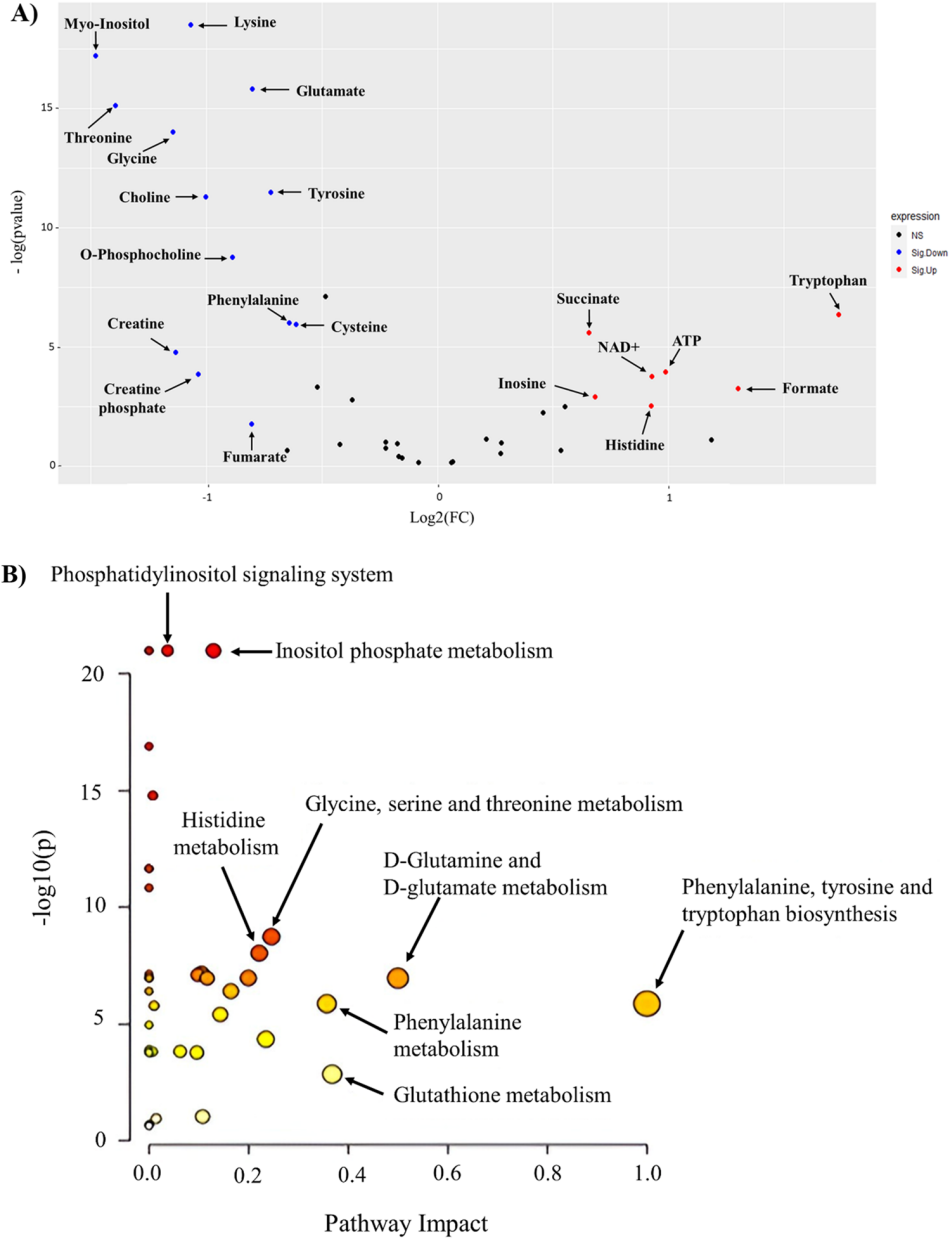

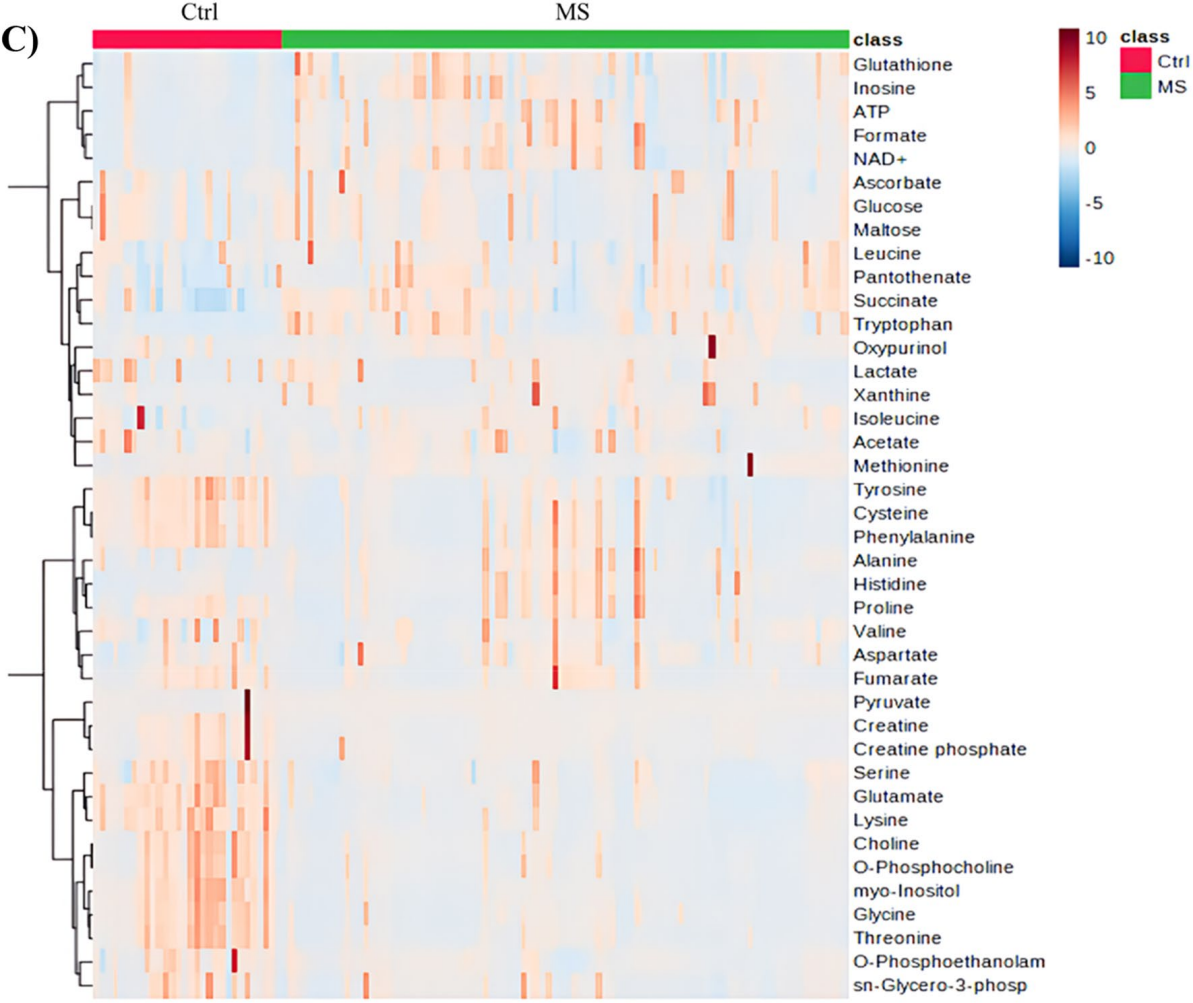
Significantly altered metabolites clusters and metabolites in patients with MS compared to healthy controls (**A**) Volcano plots of up (red) and down (blue) regulated metabolites generated by Rstudio software using raw p-value and fold change (FC) cutoffs of < 0.05 and 1.5, respectively. (**B**) Pathway analysis of metabolites in serum patients with MS compared to Ctrl. The x-axis presents the pathway impact, while the y-axis shows the most significantly altered clusters on the top. The color and size of each circle is based on the p-value and pathway impact value, respectively. (**C**) Heatmap of the 40 altered metabolites. The rows demonstrate the samples, while the lines represent the metabolites detected in the serum. The color scale (right) represents the relative expression levels of the metabolites across all samples; The higher values (red) reflect higher metabolite abundance, and lower values (blue) reflect lower abundance.

Volcano plot analysis was used to compare the MS group to a healthy control group and found that seven metabolites were upregulated (red) (formate, tryptophan, ATP, histidine, NAD + , succinate, and inosine) and thirteen metabolites (lysine, myo-inositol, glycine, threonine, choline, creatine, glutamate, O-phosphocholine, phenylalanine, tyrosine, cysteine, fumarate, and creatine phosphate) were downregulated (blue) in patients with MS versus healthy controls (Raw p-value ≤ 0.05, FC cut-off of 1.5) (Fig. 3A).

The metabolites that underwent significant changes were analyzed using pathway analysis to identify pathways that were significantly dysregulated in MS. The results of the pathway analysis revealed that inositol phosphate, glycine, serine, threonine, and histidine metabolism were found to be significantly altered in the MS group compared to the control group (Fig. 3B). The findings of the metabolic pathway analysis were displayed using a bubble plot, where in each bubble denoted a specific metabolic pathway. The x-axis presents the pathway impact, while the y-axis shows -log10 (p). The size of each bubble indicates the influence factor of the pathway analysis; the larger the bubble the higher the impact value. The color of bubbles varying from yellow to red represents different levels of significance, with red indicating a more significant difference.

**Figure 4:**
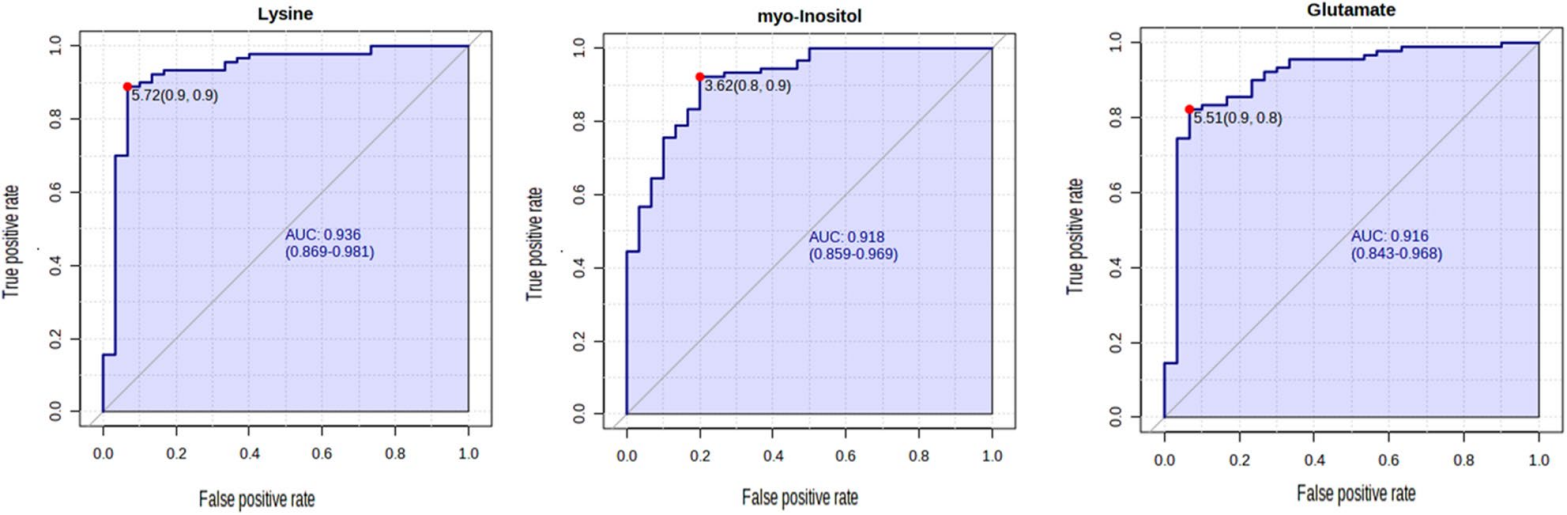
Receiver operating characteristic curves (ROC) are used to distinguish between groups MS (n = 90) and healthy group (n = 30) produced by MetaboAnalyst 5.0 software. Lysine, myo-inositol, and glutamate exhibited the highest discriminatory power more than 0.9.

The heat map in Fig. 3C presents a visual representation of the 40 altered metabolites across MS and control groups. Highlighting the differences in the intensity and distribution of different metabolites. Higher values (red) indicate increased metabolite abundance, whereas lower values (blue) indicate decreased abundance (Fig. 4).

**Figure 5:**
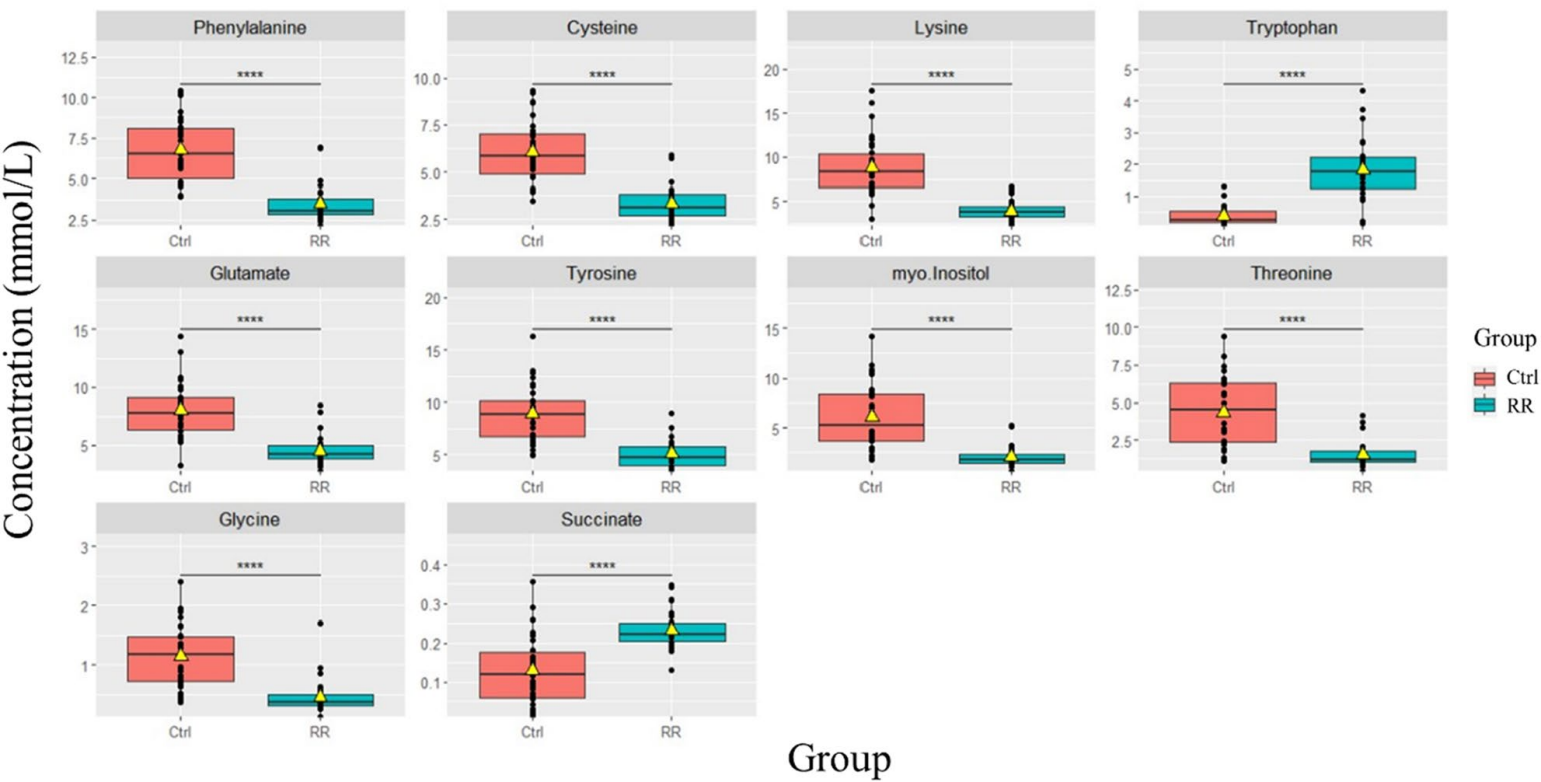
Box plots for 10 significantly altered metabolites between healthy control (n = 30) and RRMS groups (n = 30) analyzed by t-test, p-value < 0.05. (****p < 0.0001).

For each significantly altered metabolite between the MS and control groups, area under the curve (AUC) was determined in Table S2. Lysine, myo-inositol, and glutamate exhibited the highest discriminatory power (0.93, 95% CI 0.869–0.981; 0.92, 95% CI 0.859–0.969; 0.91, 95% CI 0.843–0.968 respectively, indicating that the estimated cut-off points effectively distinguish between RRMS represents the early stage and the most common form of MS^5^. Moreover, a comparative analysis of metabolites between RRMS and healthy control was conducted using box plot for 10 significant metabolites (Fig. 5). Lysine, threonine, and glycine were significantly lower in RRMS compared to control while pantothenate, glutathione, and succinate were significantly higher in RRMS (Fig. S5).

### Metabolite profiling of the three different subtypes of MS; RR, PP and SP

**Figure 6:**
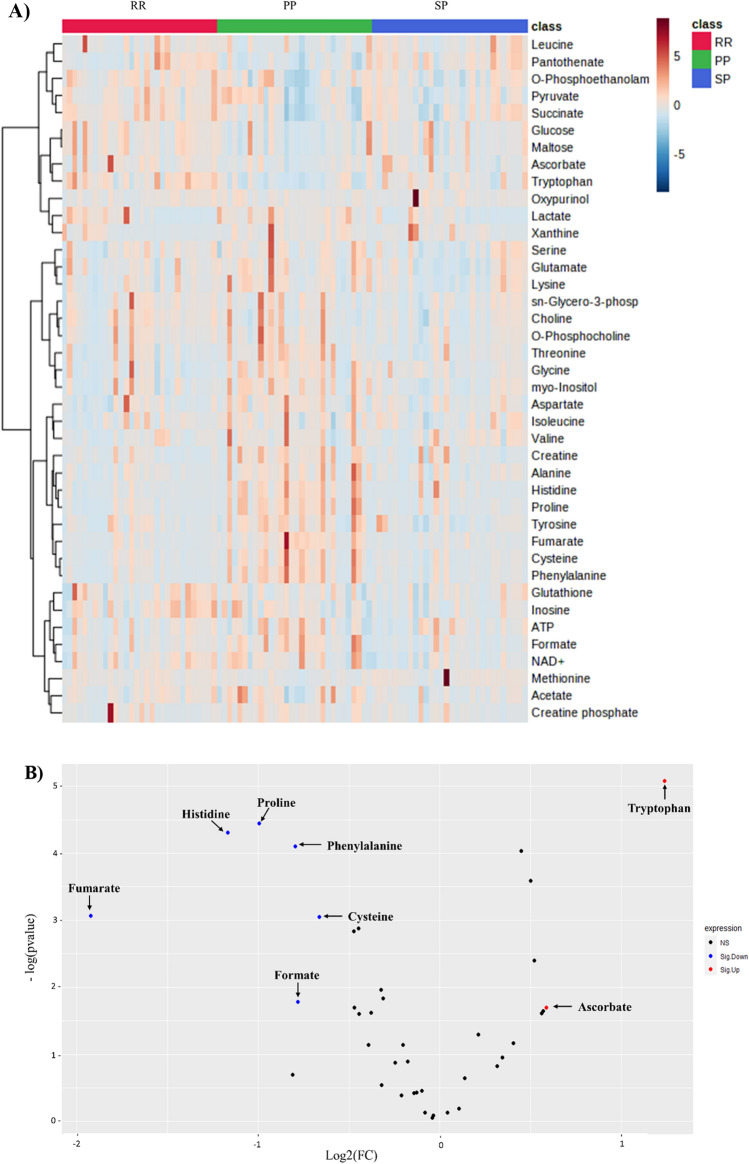

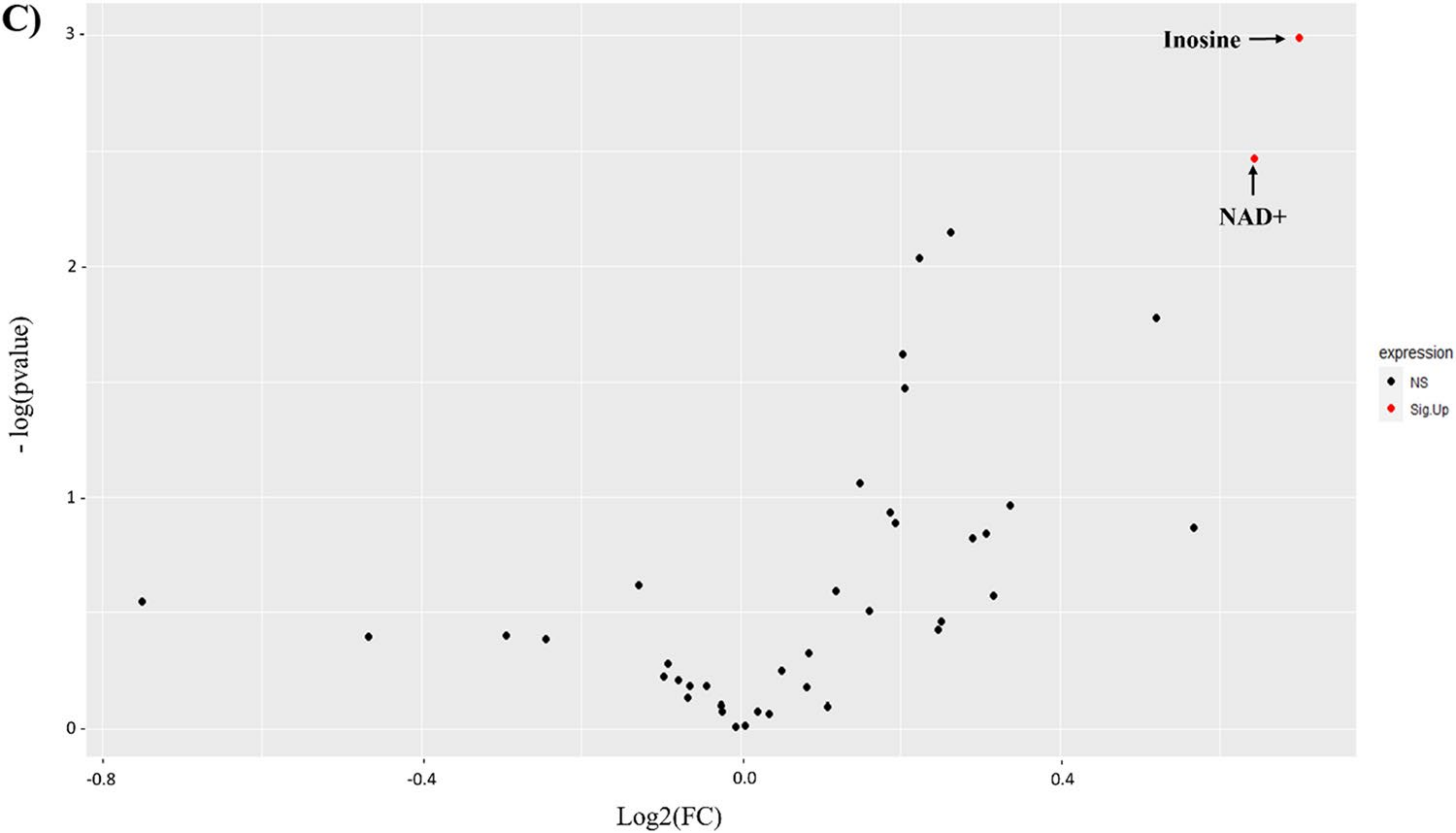
Metabolite profiling of different stages of MS; relapsing remitting (RR), primary progressive (PP), and secondary progressive (SP). (**A**) Heatmap of the top altered metabolites. The higher values (red) reflect higher metabolite abundance, and lower values (blue) reflect lower abundance produced by MetaboAnalyst 5.0 software. (**B**) Volcano plots of up (red) and down (blue) regulated metabolites in RRMS compared with PPMS generated by Rstudio software using p-value and fold change (FC) cutoffs of < 0.05 and 1.5, respectively. (**C**) Volcano plots of up (red) and down (blue) regulated metabolites in RRMS compared with SPMS generated by Rstudio software using p-value and fold change (FC) cutoffs of < 0.05 and 1.5, respectively.

Univariate data analyses were performed to elucidate alterations in metabolites across different subtypes MS. A heatmap revealed distinct metabolic patterns with differential expression among MS subtypes (Fig. 6A). In addition, the volcano plot shows eight statistically significant altered metabolites: two metabolites upregulated (red) (tryptophan, and ascorbate) and six metabolites (histidine, proline, phenylalanine, cysteine, formate, and fumarate) downregulated (blue) in RRMS compared with PPMS (Fig. 6B). While only two metabolites (inosine and NAD +) were significantly upregulated (red) in RRMS compared with SPMS (Fig. 6C).

**Figure 7:**
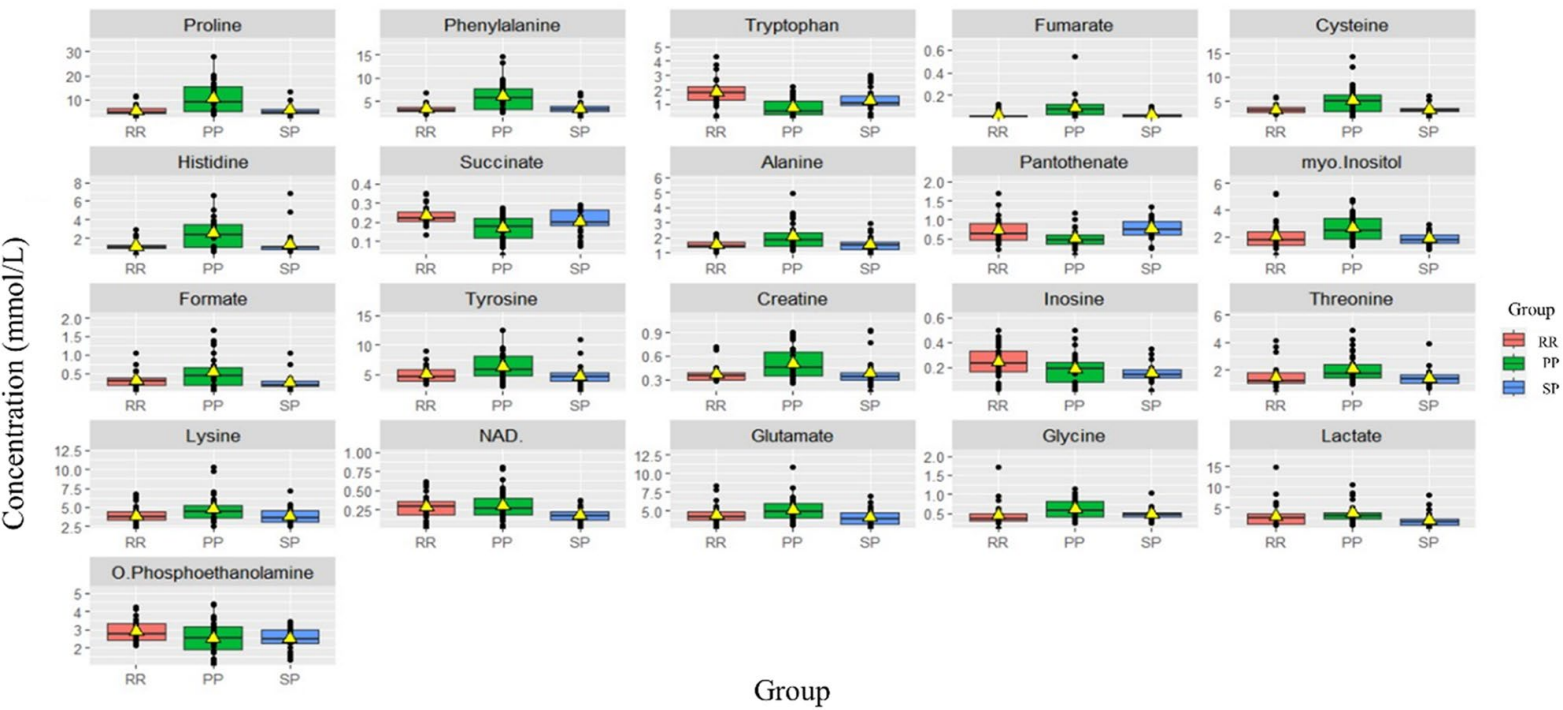
Box plots for significantly altered metabolites (n = 21) between different stages of MS; relapsing–remitting, primary progressive, and secondary progressive groups and one-way ANOVA was used.

A box plot comparing the intensities of significant metabolites across MS subtypes is presented in Fig. 7. Tryptophan and succinate significantly differed across all MS subtypes (Table 4). Inosine increased significantly among RR compared with progressive stages (PP and SP), while glutamate and lactate were significantly higher in PP than SP (Fig. 7, Table 4).

**Table 4:** Differences in levels of significant Metabolites (n = 21) detected in the three different subtypes of MS; RR, PP and SP (30 samples in each group). Significant metabolites were selected by one-way ANOVA.

Metabolites	p-value	RR vs PP	RR vs SP	PP vs SP
Proline	8.03E-07	↓*	–	↑*
Phenylalanine	1.24E-06	↓*	–	↑*
Tryptophan	1.42E-05	↑*	↑*	↓*
Fumarate	2.33E-05	↓*	–	↑*
Cysteine	3.93E-05	↓*	–	↑*
Histidine	5.48E-05	↓*	–	↑*
Succinate	4.24E-04	↑*	↑*	–
Alanine	4.93E-04	↓*	–	↑*
Pantothenate	5.55E-04	↑*	–	↓*
Myo-Inositol	1.48E-03	↓*	–	↑*
Formate	1.49E-03	↓*	–	↑*
Tyrosine	2.79E-03	↓*	–	↑*
Creatine	3.38E-03	↓*	–	↑*
Inosine	5.23E-03	–	↑*	–
Threonine	5.48E-03	↓*	–	↑*
Lysine	7.17E-03	↓*	–	↑*
NAD +	9.88E-03	–	↑*	↑*
Glutamate	1.24E-02	–	–	↑*
Glycine	2.32E-02	↓*	–	↑*
Lactate	2.53E-02	–	–	↑*
O-phosphoethanolamine	4.62E-02	–	↑*	–

“*”represents significance (p-value < 0.05) determined by t‐test using MetaboAnalyst 5.0 software.

“↑”indicates increased metabolite levels, “↓” indicates decreased metabolite levels, “–” indicated that the metabolite did not significantly change between compared groups.

Pantothenate, ascorbate, methionine, succinate, and tryptophan were significantly higher in concentrations in RR compared to PP, whereas myo-inositol, alanine, and creatine were significantly lower in RR than PP (Fig. S6 and Table S4).

Comparison of metabolites concentrations between PP and SP, box plots in Fig. 7 and S7, shows that tryptophan and pantothenate were significantly higher in levels in SPMS compared to PPMS (Table S5). According to Table S6, NAD + , inosine, and tryptophan were significantly higher in RR than in SP (Figs. 7 and S8).

## Discussion

The urgent need for the discovery of potential novel biomarkers for early diagnosis and prognosis prediction of MS disease has driven the investigation of potential biomarkers using various experimental methods. This study utilized metabolic screening to identify biomarkers with ^1^H-NMR spectroscopy by determining changes in the metabolic profiles of patients with MS and across MS subtypes compared with a control group in serum samples.

The untargeted metabolomics method employed in our study identified a total of 25 metabolites that exhibited significant alterations in patient samples with MS when compared to control samples. Furthermore, among the various subtypes of MS, 21 metabolites displayed significant changes, providing compelling evidence for the existence of a distinct dysregulated metabolic state in MS.

Inositol phosphate, histidine, glycine, serine, threonine metabolism, as well as biosynthesis of phenylalanine, tryptophan, and tyrosine were the most altered pathways between control and MS patients. Our findings indicate alterations in metabolites associated with vital pathways involved in energy balance maintenance and immune response, such as glutamate, and tryptophan metabolism, which are consistent with previous studies^32–35^.

In order to identify metabolic signatures that can serve as distinctive biomarkers for distinguishing patients with MS from those without the condition, we examined the differences in metabolite profiles in serum samples between patients with MS and healthy control. This study revealed significantly lower levels of lysine, threonine, glycine, phosphocholine, myo-inositol, and phenylalanine in the serum of MS patients compared to controls, as well as between RR and control. These findings are consistent with prior study conducted by Sylvestre et al. where they reported a correlation between alterations in phenylalanine and myo-inositol levels and phenotypic characteristics in patients with MS^36^. Moreover, a reduction in phenylalanine levels was associated with higher expanded disability status scale scores^33^. In contrast, myo-inositol levels were higher in CSF from patients with RRMS compared to control. Inositol phosphate and myo-inositol are constituents of myelin, playing significant roles in neural function and homeostasis within the CNS^37,38^. Therefore, the concentrations of serum myo-inositol may serve as indicator of disruptions in inositol phosphate metabolism due to demyelination^39^.

Serum glycine concentration was significantly reduced in patients with MS and RR compared to control. Glycine, a main inhibitory neurotransmitter in the brain, has anti-inflammatory effects by regulating immune cell functions on a systemic level^40^. Therefore, the decrease in glycine concentration may be linked to an imbalance between pro-inflammatory versus anti-inflammatory mediators in MS disease. Concentrations of glutathione, formate, inosine, succinate, and pantothenate were observed to be higher in RR compared to control as well as in MS compared to control.

Succinate is a metabolic intermediate in the tricarboxylic acid (TCA) cycle that contributes to inflammation leading to increase their levels in inflammatory-related health conditions such as MS^41^. Elevated serum succinate levels could suggest an increased energy demand in demyelinated axons, combined with mitochondria dysfunction, and these thought to be the root causes of the neurodegenerative process in MS^42^. Furthermore, succinate levels were found to be elevated in urine samples from patients with MS^43^.

Our findings indicated that formate levels in RRMS were higher compared to the control group, which aligns with the results reported by Yeo et al.^44^ reflecting perturbed energy metabolism in MS.

In addition, a targeted metabolomics of 102 metabolites in serum samples analyzed by mass spectrometry-based methods, revealed increased pantothenate levels in patients with MS compared to control which is consistent with our findings^45^. Pantetheine is an intermediate in coenzyme A biosynthesis, plays a crucial role in energy metabolism, particularly in mitochondrial reactions^46^. Thus, this result supports the explanation in disturbances of energy metabolism in MS disease.

Glutamate has shown high diagnostic accuracy in discriminating MS from healthy cases. Our results are in agreement with a GC–MS-based metabolomic study that showed that serum glutamate could discriminate the profile of MS from healthy control^47^. The accumulation of glutamate in patients with MS is linked to axon damage, driving various symptoms^48^.

We observed decreased levels of choline in MS compared to control, which agreed with previous studies conducted in CSF samples^49,50^. However, the results in this study were inconsistent with previous studies that show decreased choline levels in blood samples from patients with MS^32^. The lower values of choline with progression of MS may be attributed to muscle weakness and damage^51^.

## Conclusion

In summary, our study has identified a differentiation of distinct metabolic profiles between patients diagnosed with MS and healthy controls, as well as across MS subtypes identified by ^1^H-NMR spectroscopy. Univariate and multivariate analyses revealed a number of metabolites in serum that exhibited significant alterations between the MS group and the control group. In addition, lysine, myo-inositol, and glutamate exhibited the highest discriminatory power between healthy control and MS according to AUC results. Our findings shed light on the extent of metabolome changes in MS, potentially aiding in the identification of biomarkers for disease diagnosis and monitoring. Future research will focus on assessing the ability of lysine, myo-inositol, and glutamate, along with other reported potential biomarkers, to differentiate between MS patients and healthy controls in a larger cohort. Additionally, metabolites that exhibited a trend (either an increase or decrease) in their levels with disease progression, such as inosine and O-phosphoethanolamine, will be validated in a larger cohort using more sensitive approaches (e.g. targeted mass spectrometry) to ensure the robustness and reliability of these metabolites in distinguishing different MS subtypes.

### Supplementary Information

Supplementary Information.

## Data Availability

The datasets used and/or analysed during the current study available from the corresponding author on reasonable request.
