# Supplementary material for: NMR-based metabolomics identification of potential serum biomarkers of disease progression in patients with multiple sclerosis
Source: Sci Rep. 2024 Jun 26;14:14806. doi: 10.1038/s41598-024-64490-x (PMC11208524; doi:10.1038/s41598-024-64490-x)
Supplement: Supplementary file 1 — Supplementary Information. [file 41598_2024_64490_MOESM1_ESM.docx]

**Supplementry figures:**


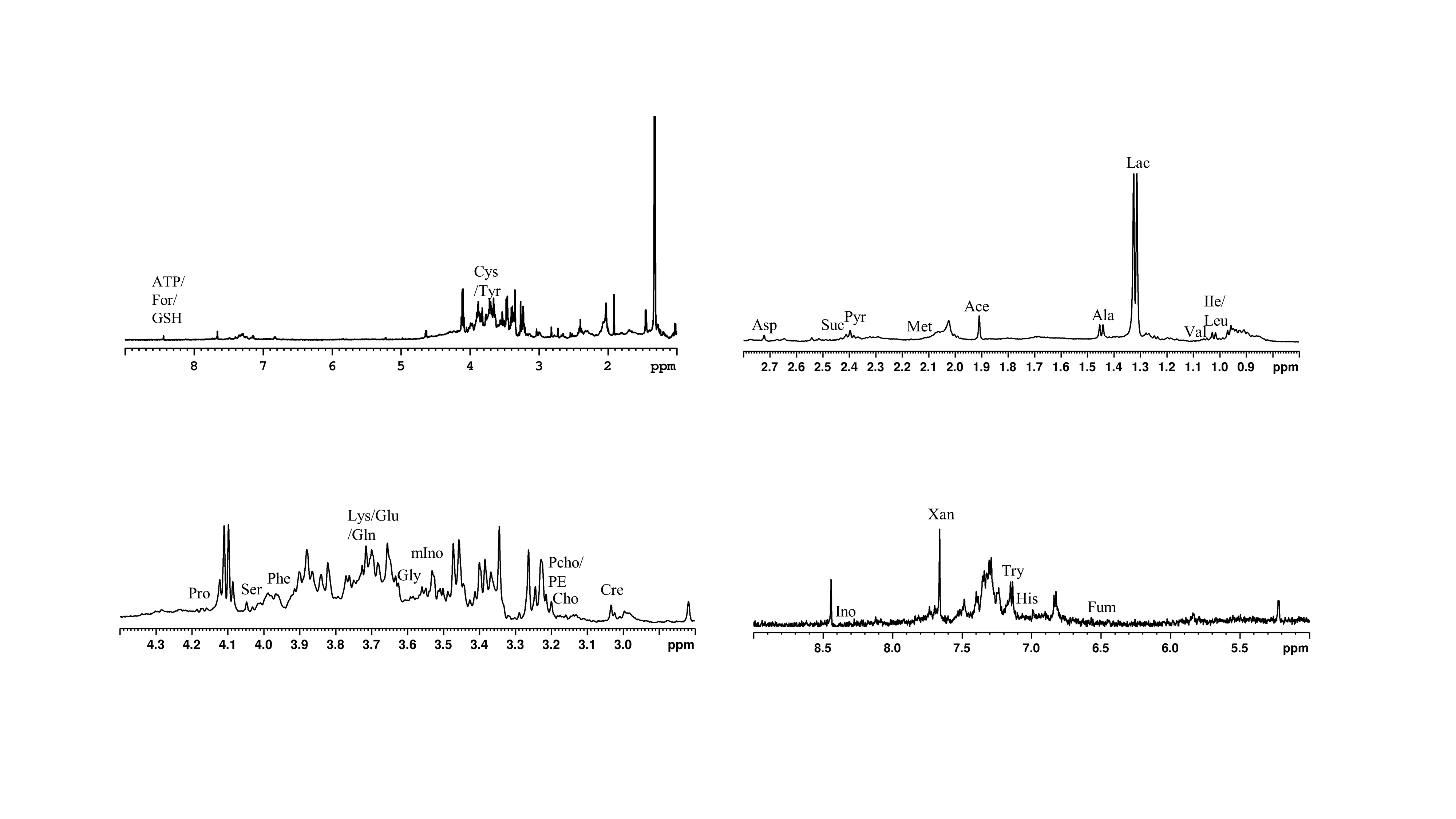


**Figure S1.** Representative 1H-NMR spectrum of a control serum sample. Full spectrum (upper left) and three expansions. Abbreviations: Ace: acetate, Ala: alanine, Asp: aspartate, ATP: adenosine triphosphate, Cho: choline, Cre: creatine, Cys: Cysteine, For: formate, Fum: fumarate, Glu: glutamate, Gln: glutamine, GSH: glutathione, Gly: glycine, His: histidine, IIe: isoleucine, Ino: inosine, Lac: lactate, Leu: leucine, Lys: lysine, Met: methionine, mIno: myo-inositol; PCho: O-phosphocholine, PE: O-phosphoethanolamine, Phe: phenylalanine; Pro: proline, Pyr: pyruvate, Ser: serine, Suc: succinate, Try: Tryptophan, Tyr: tyrosine, Val: valine, Xan: xanthine.


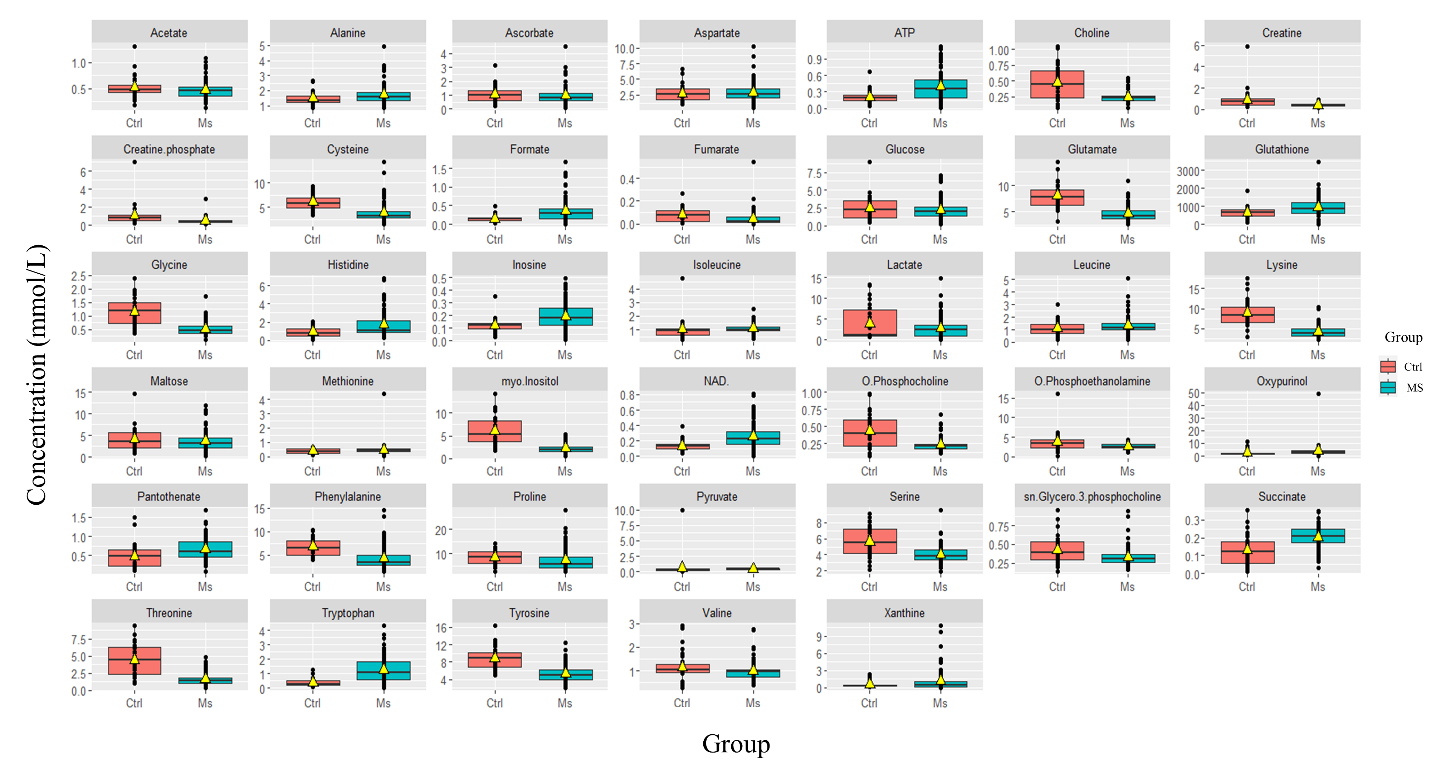


**Figure S2.** Boxplots showing the concentrations (y-axis) of the all metabolites (n=40) found in control (n=30) vs MS groups (n=90) analyzed by t-test, p-value <0.05 (Table S1). The black bars show the respective median of a distribution, while the yellow triangles display the respective average. Please note that the scale of the y-axis was adapted to the concentration range and is therefore different among the different metabolites.


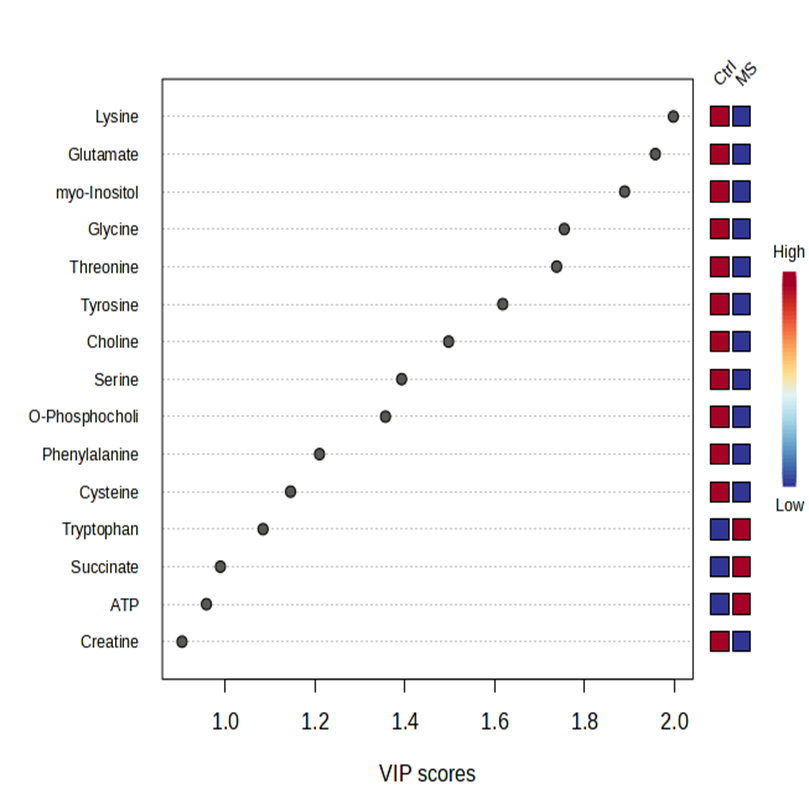


**Figure S3.** Variables Importance of Projection (VIP) plot based on OPLS-DA model using Metaboanalyst 5.0 software identifying the top 15 metabolites contributing to the difference between MS samples (n=90) vs. control samples (n=30). Twelve metabolites with a VIP score close to or greater than 1 can be considered important in a given model. The x-axis represents the VIP score, and the y-axis represents the metabolites.

**
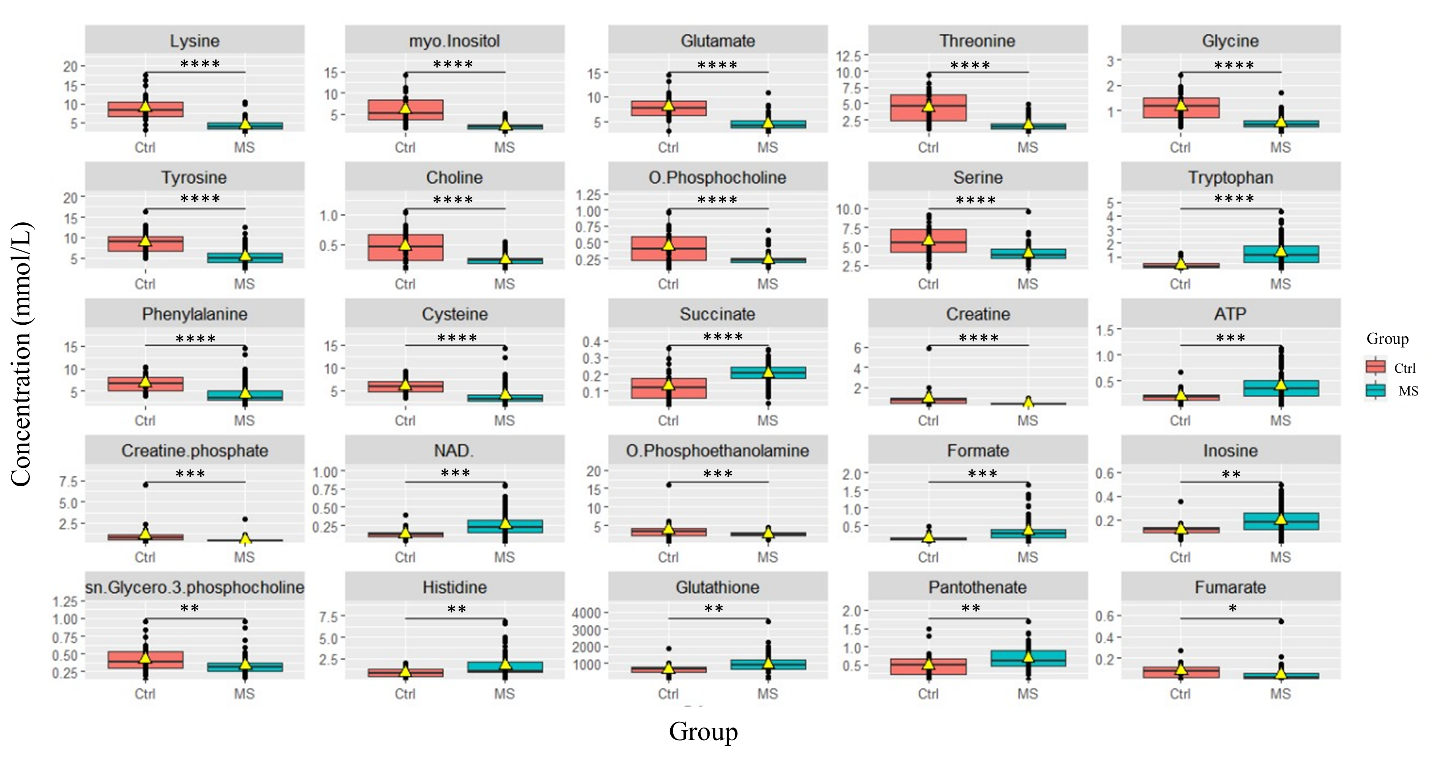
**

**Figure S4.** Boxplots showing the concentrations (y-axis) of the significant metabolites (n=25) found in control (n=30) vs MS groups (n=90) analyzed by t-test, p-value <0.05 (Table S1). The black bars show the respective median of a distribution, while the yellow triangles display the respective average. Please note that the scale of the y-axis was adapted to the concentration range and is therefore different among the different metabolites. (*p< 0.05, **p< 0.01, ***p< 0.001, and ****p < 0.0001)


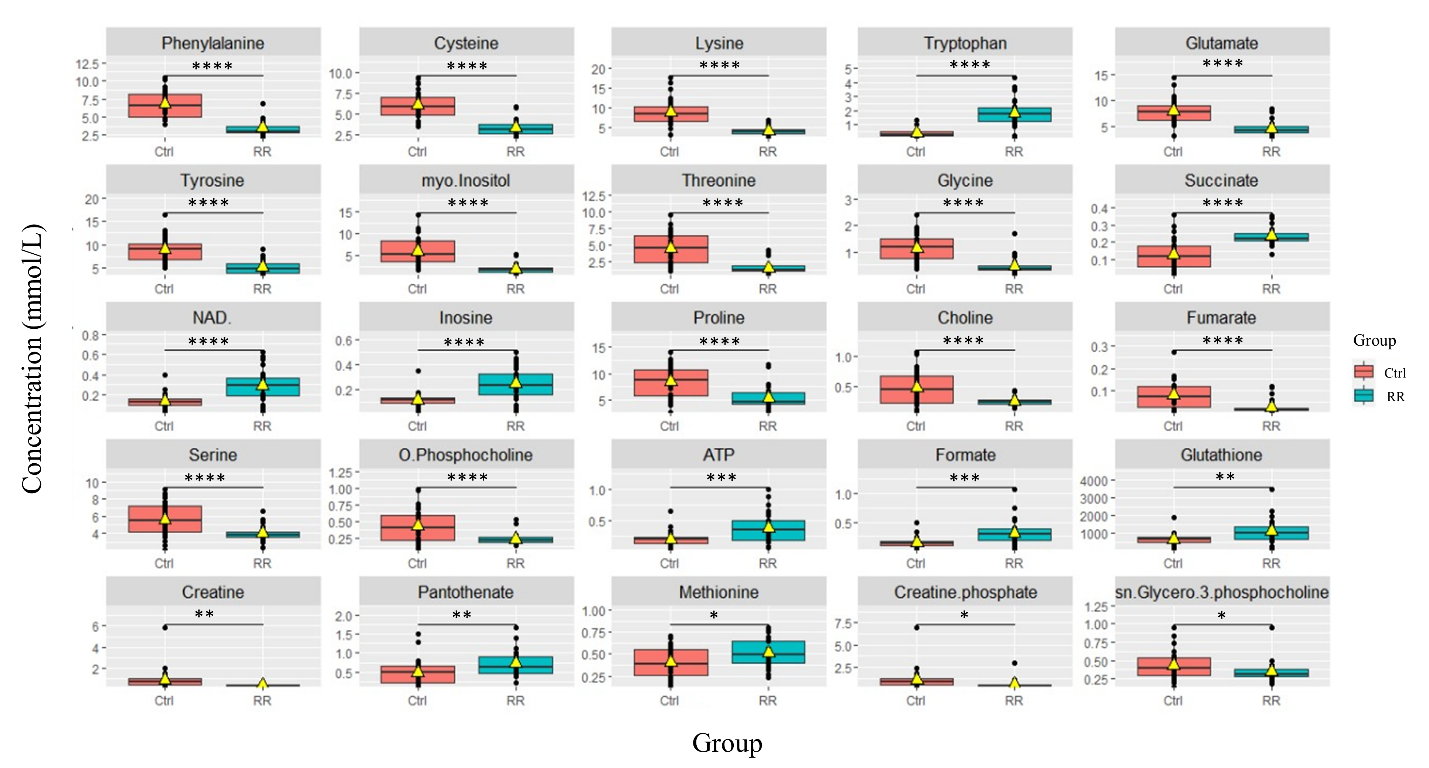


**Figure S5.** Boxplots showing the concentrations (y-axis) of the significant metabolites (n=25) found in control (n=30) vs RRMS group (n=30) analyzed by t-test, p-value <0.05 (Table S3). The black bars show the respective median of a distribution, while the yellow triangles display the respective average. Please note that the scale of the y-axis was adapted to the concentration range and is therefore different among the different metabolites. (*p< 0.05, **p< 0.01, ***p< 0.001, and ****p < 0.0001)


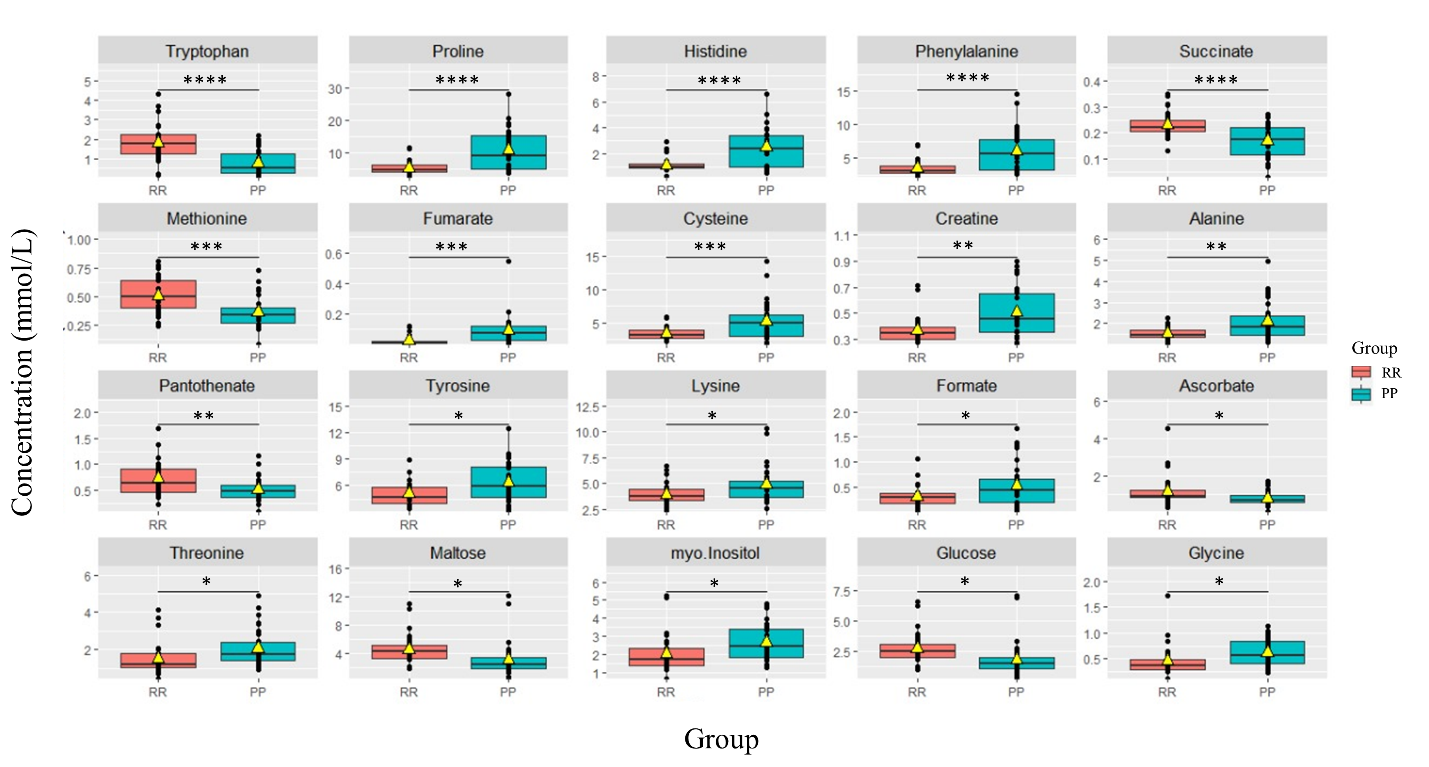


**Figure S6.** Boxplots showing the concentrations (y-axis) of the significant metabolites (n=20) found in RRMS (n=30) vs. PPMS groups (n=30) analyzed by t-test, p-value <0.05 (Table S5). The black bars show the respective median of a distribution, while the yellow triangles display the respective average. Please note that the scale of the y-axis was adapted to the concentration range and is therefore different among the different metabolites. (*p< 0.05, **p< 0.01, ***p< 0.001, and ****p < 0.0001)

**
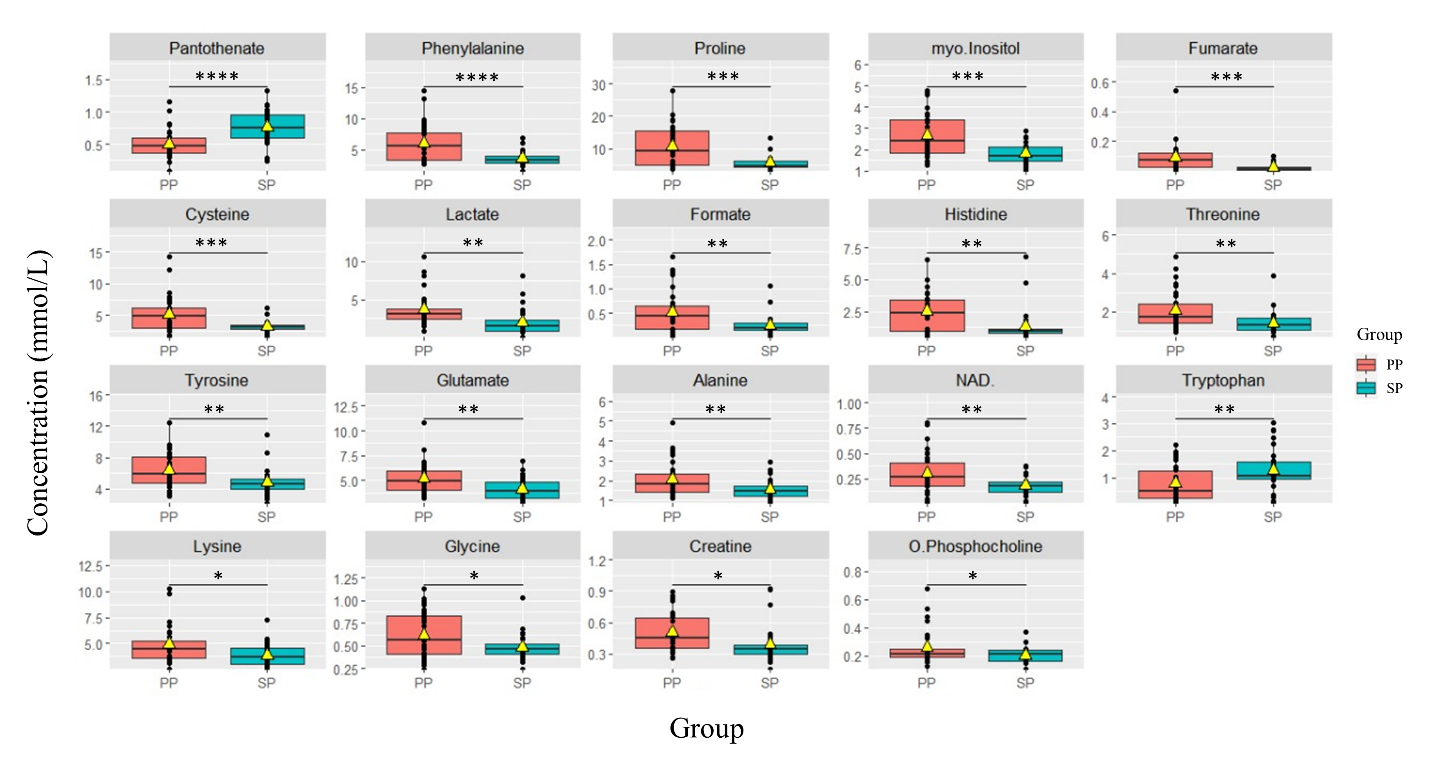
Figure S7.** Boxplots showing the concentrations (y-axis) of the significant metabolites (n=19) found in PPMS (n=30) vs. SPMS groups (n=30) analyzed by t-test, p-value <0.05 (Table S6). The black bars show the respective median of a distribution, while the yellow triangles display the respective average. Please note that the scale of the y-axis was adapted to the concentration range and is therefore different among the different metabolites. (*p< 0.05, **p< 0.01, ***p< 0.001, and ****p < 0.0001)

**
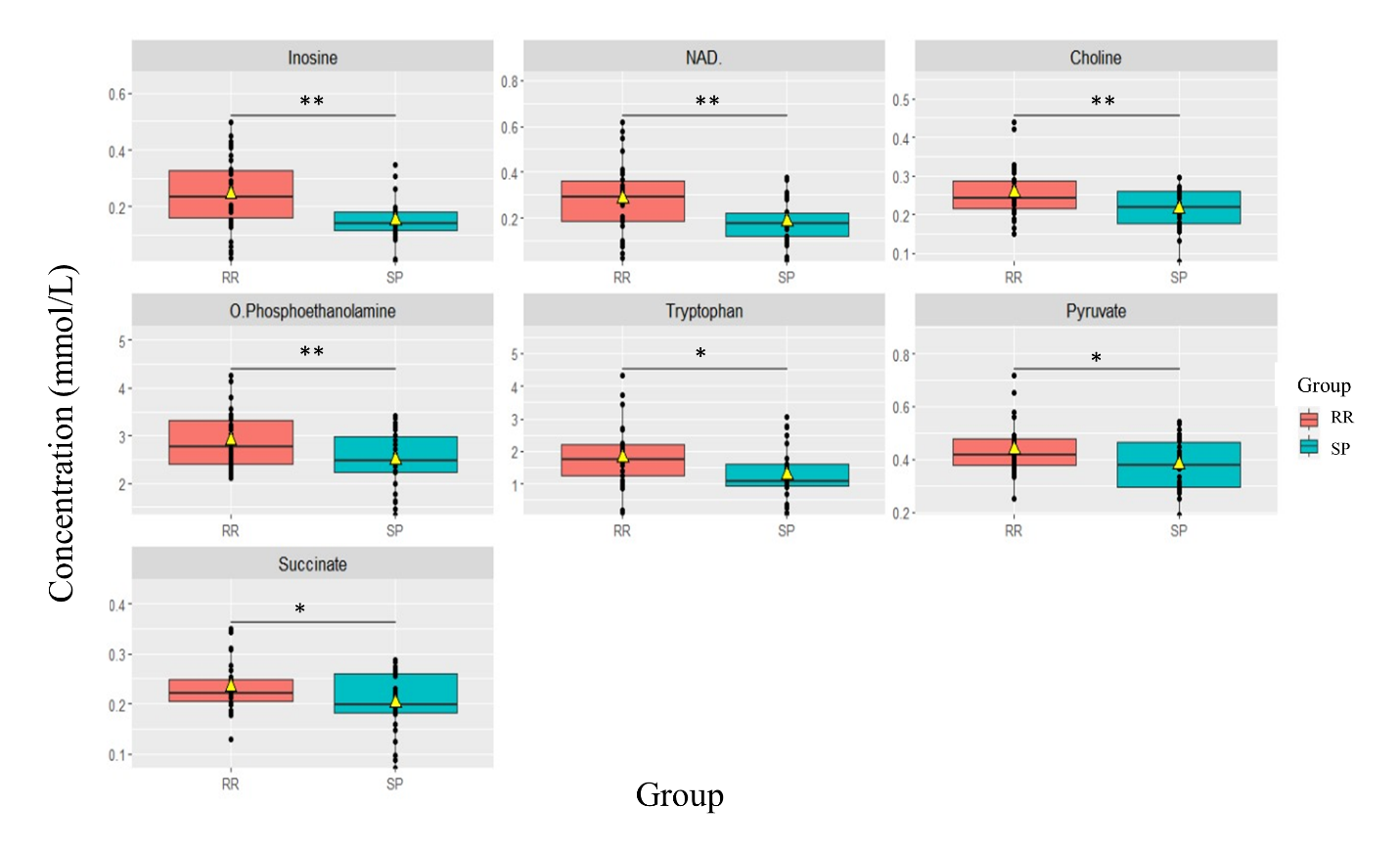
**

**Figure S8.** Boxplots showing the concentrations (y-axis) of the significant metabolites (n=7) found in RRMS (n=30) vs. SPMS groups (n=30) analyzed by t-test, p-value <0.05 (Table S7). The black bars show the respective median of a distribution, while the yellow triangles display the respective average. Please note that the scale of the y-axis was adapted to the concentration range and is therefore different among the different metabolites. (*p < 0.05 and **p< 0.01)

**Supplementry tables:**

**Table S1:** Metabolites (n=40) detected in MS samples (n=90) vs healthy samples (n=30) using cryogenic probe NMR spectroscopy. Data processing was done as described in the methodological part. P‐values are used to compare metabolite levels in the MS samples and healthy group. P‐values for metabolites with significantly different levels between the two groups are shown in bold. P‐values were determined by t‐test using Metaboanalyst 5.0 software. (*p< 0.05, **p< 0.01, ***p< 0.001, and ****p < 0.0001)

| ***Metabolites*** | ***p‐value*** | ***Metabolites*** | ***Columnp‐value4*** |
| --- | --- | --- | --- |
| **Lysine** | **3.11E-19 ****** | **sn-glycero-3-phosphocholine** | **1.67E-03 **** |
| **myo-Inositol** | **6.13E-18 ****** | **Histidine** | **2.90E-03 **** |
| **Glutamate** | **1.56E-16 ****** | **Glutathione** | **3.29E-03 **** |
| **Threonine** | **7.64E-16 ****** | **Pantothenate** | **5.54E-03 **** |
| **Glycine** | **9.51E-15 ****** | **Fumarate** | **1.76E-02 *** |
| **Tyrosine** | **3.33E-12 ****** | Alanine | 7.42E-02 |
| **Choline** | **5.18E-12 ****** | Xanthine | 7.90E-02 |
| **O-phosphocholine** | **1.79E-09 ****** | Valine | 9.60E-02 |
| **Serine** | **7.47E-08 ****** | Leucine | 1.09E-01 |
| **Tryptophan** | **4.39E-07 ****** | Acetate | 1.12E-01 |
| **Phenylalanine** | **9.54E-07 ****** | Lactate | 1.21E-01 |
| **Cysteine** | **1.10E-06 ****** | Proline | 1.82E-01 |
| **Succinate** | **2.50E-06 ****** | Pyruvate | 2.18E-01 |
| **Creatine** | **1.64E-05 ****** | Oxypurinol | 2.27E-01 |
| **ATP** | **1.11E-04 ***** | Methionine | 3.00E-01 |
| **Creatine phosphate** | **1.39E-04 ***** | Maltose | 3.86E-01 |
| **NAD+** | **1.77E-04 ***** | Glucose | 4.46E-01 |
| **O-phosphoethanolamine** | **4.79E-04 ***** | Isoleucine | 6.32E-01 |
| **Formate** | **5.65E-04 ***** | Ascorbate | 6.78E-01 |
| **Inosine** | **1.24E-03 **** | Aspartate | 7.00E-01 |

| ***Metabolites*** | ***AUC*** | ***Metabolites*** | ***A*** ***AUC AUC*** |
| --- | --- | --- | --- |
| Lysine | 0.93111 | Histidine | 0.69926 |
| Myo-Inositol | 0.91593 | Pantothenate | 0.68407 |
| Glutamate | 0.9137 | Fumarate | 0.68037 |
| Glycine | 0.87185 | Sn-glycero-3-phosphocholine | 0.67963 |
| Tyrosine | 0.87074 | Proline | 0.67481 |
| Threonine | 0.87037 | Oxypurinol | 0.67333 |
| Cysteine | 0.85148 | O-phosphoethanolamine | 0.6663 |
| Phenylalanine | 0.83704 | Pyruvate | 0.62185 |
| Tryptophan | 0.8137 | Isoleucine | 0.61926 |
| Serine | 0.76704 | Alanine | 0.61407 |
| Choline | 0.76444 | Valine | 0.60259 |
| Formate | 0.76074 | Leucine | 0.59852 |
| ATP | 0.76 | Acetate | 0.59593 |
| Succinate | 0.75778 | Methionine | 0.57741 |
| Creatine | 0.75593 | Ascorbate | 0.57037 |
| NAD+ | 0.75 | Maltose | 0.55852 |
| Creatine phosphate | 0.74778 | Xanthine | 0.55741 |
| O-phosphocholine | 0.74148 | Glucose | 0.55 |
| Inosine | 0.72111 | Lactate | 0.5263 |
| Glutathione | 0.70148 | Aspartate | 0.51444 |

**Table S2:** “Area under the curve” (AUC) values obtained from receiver operating characteristic (ROC) curves analysis based on the metabolite levels determined in the 90 samples of MS and 30 healthy samples analyzed in this study. Three of the 40 metabolites, namely Lysine, myo-Inositol, and Glutamate showed an AUC value higher than 0.9.

**Table S3:** The significant Metabolites (n=25) detected in RRMS samples (n=30) vs healthy samples (n=30) using cryogenic probe NMR spectroscopy. p‐values are used to compare metabolite levels in the RRMS samples and healthy group. p‐values were determined by t‐test using Metaboanalyst software.

| ***Metabolites*** | ***p-value*** | ***Metabolites*** | ***p-value*** |
| --- | --- | --- | --- |
| Phenylalanine | 6.21E-11 | Choline | 3.63E-05 |
| Cysteine | 7.97E-11 | Fumarate | 3.93E-05 |
| Lysine | 1.22E-10 | Serine | 5.88E-05 |
| Tryptophan | 1.42E-10 | O-phosphocholine | 8.58E-05 |
| Glutamate | 1.34E-09 | ATP | 3.50E-04 |
| Tyrosine | 5.07E-09 | Formate | 4.26E-04 |
| Myo-Inositol | 2.27E-08 | Glutathione | 1.40E-03 |
| Threonine | 3.19E-08 | Creatine | 4.76E-03 |
| Glycine | 3.78E-08 | Pantothenate | 6.77E-03 |
| Succinate | 4.74E-07 | Methionine | 2.73E-02 |
| NAD+ | 9.35E-06 | Creatine phosphate | 3.04E-02 |
| Inosine | 1.38E-05 | Sn-glycero-3-phosphocholine | 4.85E-02 |
| Proline | 1.87E-05 |  |  |

**Table S4:** The significant Metabolites (n=20) detected in RRMS samples (n=30) vs PPMS samples (n=30) using cryogenic probe NMR spectroscopy. P‐values are used to compare metabolite levels in the RRMS samples and PPMS group. P‐values were determined by t‐test using Metaboanalyst 5.0 software.

| ***Metabolites*** | ***p-value*** | ***Metabolites*** | ***p-value*** |
| --- | --- | --- | --- |
| Tryptophan | 8.38E-06 | Formate | 1.65E-02 |
| Proline | 3.59E-05 | Ascorbate | 2.00E-02 |
| Histidine | 4.92E-05 | Threonine | 2.02E-02 |
| Phenylalanine | 7.95E-05 | Maltose | 2.24E-02 |
| Succinate | 9.20E-05 | Myo-Inositol | 2.41E-02 |
| Methionine | 2.59E-04 | Glucose | 2.45E-02 |
| Fumarate | 8.66E-04 | Glycine | 2.47E-02 |
| Cysteine | 8.90E-04 |  |  |
| Creatine | 1.32E-03 |  |  |
| Alanine | 1.47E-03 |  |  |
| Pantothenate | 4.02E-03 |  |  |
| Tyrosine | 1.08E-02 |  |  |
| Lysine | 1.46E-02 |  |  |

| ***Metabolites*** | ***p-value*** | ***Metabolites*** | ***p-value*** |
| --- | --- | --- | --- |
| Pantothenate | 7.64E-05 | Tyrosine | 3.62E-03 |
| Phenylalanine | 9.86E-05 | Glutamate | 5.11E-03 |
| Proline | 1.10E-04 | Alanine | 5.57E-03 |
| Myo-Inositol | 1.83E-04 | NAD+ | 6.43E-03 |
| Fumarate | 5.20E-04 | Tryptophan | 8.78E-03 |
| Cysteine | 7.77E-04 | Lysine | 1.09E-02 |
| Lactate | 1.75E-03 | Glycine | 1.59E-02 |
| Formate | 2.34E-03 | Creatine | 1.71E-02 |
| Histidine | 2.83E-03 | O-phosphocholine | 3.57E-02 |
| Threonine | 3.12E-03 |  |  |

**Table S5:** The significant Metabolites (n=19) detected in PPMS samples (n=30) vs SPMS samples (n=30) using cryogenic probe NMR spectroscopy. P‐values are used to compare metabolite levels in the RRMS samples and PPMS group. P‐values were determined by t‐test using Metaboanalyst 5.0 software.

**Table S6:** The significant Metabolites (n=7) detected in RRMS samples (n=30) vs SPMS samples (n=30) using cryogenic probe NMR spectroscopy. P‐values are used to compare metabolite levels in the RRMS samples and PPMS group. P‐values were determined by t‐test using Metaboanalyst 5.0 software.

| ***Metabolites*** | ***p-value*** |
| --- | --- |
| Inosine | 1.02E-03 |
| NAD+ | 3.40E-03 |
| Choline | 7.11E-03 |
| O-phosphoethanolamine | 9.17E-03 |
| Tryptophan | 1.68E-02 |
| Pyruvate | 2.39E-02 |
| Succinate | 3.38E-02 |
